# Environmental Legacy Across the Lifespan: Eco-Generativity, Nature Exposure, and Pro-Environmental Behavior

**DOI:** 10.1093/geroni/igaf122.1265

**Published:** 2025-12-31

**Authors:** Selma Korlat, Mathew White, Jana Nikitin

**Affiliations:** University of Vienna, Vienna, Wien, Austria; University of Vienna, Vienna, Wien, Austria; University of Vienna, Vienna, Wien, Austria

## Abstract

Generativity and the legacy one leaves behind have been proposed as strong developmental motivators for older adults to engage in actions that mitigate the negative effects of climate change (Diehl, 2022). However, only a handful of studies have specifically examined social generativity (care for people) and eco-generativity (care for nature) as distinct motivations for preserving humankind and its resources. Moreover, previous research suggests that nature exposure promotes prosocial and environmentally sustainable behaviors. The goal of this study was to investigate the facilitating role of nature exposure in social and eco-generativity across the lifespan. In Study 1, data from a nationally representative survey in the UK were analyzed (N = 18,633). The results showed that, for older adults, nature exposure was more strongly associated with social generativity, whereas the relationship between nature exposure and eco-generativity did not vary significantly across age groups. Study 2 followed up on these findings, investigating whether momentary nature exposure increases social and eco-generativity, and whether these effects translate into pro-environmental action across the lifespan. Results from an experience sampling study (N = 103, Mage = 47.53, SDage = 17.96, age-range = 19–79) showed that momentary nature exposure increases both momentary social and eco-generativity and provided support for a partial mediation of eco-generativity in the relationship between momentary nature exposure and momentary pro-environmental behavior across the lifespan. Using different approaches and study scope, both studies consistently highlight the benefits of nature exposure in fostering care for environmental preservation and encouraging pro-environmental action in all age groups.

